# Mental health memes: beneficial or aversive in relation to psychiatric symptoms?

**DOI:** 10.1057/s41599-022-01381-4

**Published:** 2022-10-13

**Authors:** Umair Akram, Jennifer Drabble

**Affiliations:** 1 Department of Psychology, Sociology and Politics, Sheffield Hallam University, Sheffield, UK; 2 School of Psychology, University of Lincoln, Lincoln, UK

**Keywords:** Psychology, Cultural and media studies

## Abstract

Composed of an image and short caption, internet memes visually depict an element of a culture or behavioural system, in a humorous way that contextually relates to a particular demographic. Typically, they are rapidly shared, with many variations of the original. Online interaction with internet memes has become a crucial psychosocial aspect of digital culture, which have recently become well established in popular media by consistently maintaining culturally topical and socially salient references. Increasingly, many pages are dedicated to sharing memes related to the symptom experience of specific psychiatric disorders. Despite their popularity, the individual motivation for the observation and sharing of mental health memes remains poorly understood. While several psychiatrists and media outlets perceive internet memes related to mental health difficulties to be associated with adverse consequences, the empirical evidence fails to support this notion. Among individuals experiencing psychiatric difficulties, we explore whether interacting with mental health memes involves adverse consequences, or rather serve as a beneficial coping mechanism. Here, evaluation of the literature indicates that most psychiatrically vulnerable individuals report positive experiences when engaging with such memes. More specifically, they are perceived to facilitate a humorous take on a negative experience and situation, and the perception of peer-support through social bonds with others experiencing similar symptoms. While mental health memes typically depict dark and negative humour, their proximal nature to those experiencing psychiatric symptoms may be considered contextually positive. As such, to conclude, we discuss the role of contextual humour in facilitating cognitive reappraisal of negative thoughts and experiences. Furthermore, we set an agenda to address key methodological limitations of existing work while providing suggestions for future research.

## Introduction

Evolutionary biologist Richard Dawkins (1976) first introduced the term meme, based on the notion that individuals utilise creativity to conceptually manipulate an idea to the extent where it is mutated and subsequently spread through derivatives (Shifman, 2013). Dawkins considered memes equivalent to biological genes, whereby both transfer information between individuals while also transmitting and spreading though derivatives. Before the internet, Dawkins (1976) conceptualisation of memes encompassed ideas, behaviours, skills, verbal phrases, and fashion styles communicated either: visually; verbally; or electronically (Hernández-Cuevas and Cruz-Bermudez, 2021).

**Fig. 1:**
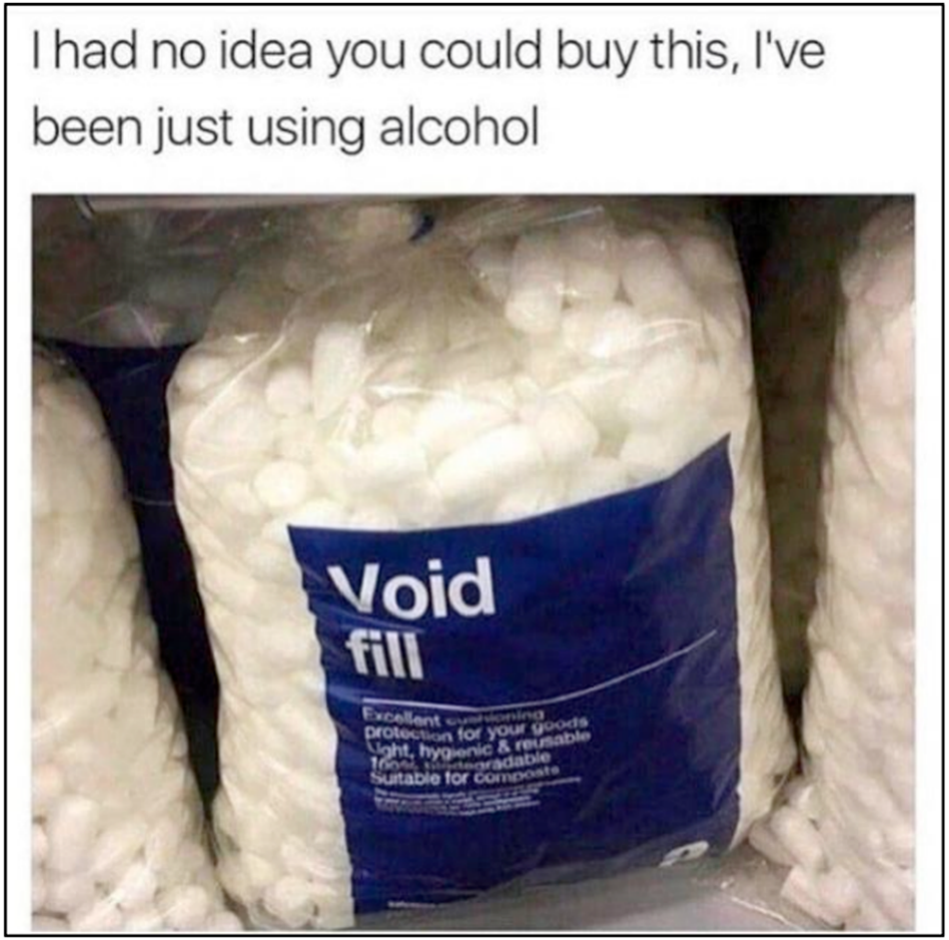
Example of a mental health meme related to depression. Image gathered from the public domain each marked with either the Public Domain Mark 1.0 or CC0 1.0 Universal.

In recent years, internet memes have gained significant popularity when considering the ever-increasing popularity of social media platforms and online forums. While conceptually like traditional memes, yet differing slightly, contemporary internet memes visually depict an element of a culture or behavioural system, in a humorous way that contextually relates to a particular demographic (Davison, 2012). Typically, internet memes are comprised of an image and short caption, which are rapidly shared, usually with slight variations of the original. Internet memes have become a crucial aspect of digital culture that are well established in the media by consistently maintaining topical and socially salient references often stepping into cultural and political domains (Shifman, 2013). Increasingly, many pages are dedicated to sharing memes related to the symptom experience of specific psychiatric disorders (e.g., anxiety, depression, addition, borderline personality, suicidal ideation: see Fig. 1 for example). Indeed, online social media pages and forums dedicated to mental health memes are often comprised of a large user base. For example, the online forum Reddit hosts a subreddit named *memes and misery* followed by ninety-five thousand individuals, with another called *depression memes* followed by approximately over two-hundred thousand individuals.

The observation and sharing of mental health memes remain poorly understood, from motivating factors to the nature of content depicted. In the present paper, we address claims that mental health memes perpetuate the specific symptom severity of an individual’s difficulties, expose vulnerable individuals to explicit content, while also encouraging adverse behaviour (Brown, 2019; Dubicka and Theodosiou, 2020; Harley, 2022; Morgan, 2021). Additionally, we explore the possibility that internet memes related to mental health difficulties may serve as a beneficial coping mechanism for some.

## Key concerns

Recently, under the premise that they graphically depict and promote adverse behaviour, the Royal College of Psychiatrists (UK) deemed mental health memes to be a social contagion^1^ and threat to public health (Dubicka and Theodosiou, 2020). Subsequently calling for social media platforms to provide academics with access to user data to examine potential adverse effects related to prolonged use and specific content including memes. However, this assumption of an imminent threat to public health emerged in the absence of empirical evidence. Nevertheless, this assumption has potentially skewed the widely discussed perception of mental health memes in the press, where vilification of internet memes continues despite diminutive evidence. For example, media opinion pieces entitled “Sharing mental health Memes Is making things worse, not better” (Refinery 29: Morgan, 2021), “Are therapy memes doing more harm than good?” (Body and Soul: Harley, 2022), “Are depression memes making you more depressed?” (Rewire: Brown, 2019).

These wide-reaching opinion pieces include interviews with a member of the public alongside a professional expert with a distal understanding of the topic, who with the journalist, propose additional concerns. In particular: the dark and symptom specific nature of mental health memes; interacting with memes rather than seeking social support or clinical treatment; the notion that observing mental health memes perpetuate ruminative thinking (Brown, 2019; Dubicka and Theodosiou, 2020; Harley, 2022; Morgan, 2021).

While these concerns are certainly understandable, recent social media analysis of images tagged as self-harm failed to demonstrate encouragement of such behaviour in other people (Shanahan et al., 2019). Indeed, explicit representation of self-harming behaviour itself (e.g., cutting) appeared only in personal posts rather than depression related memes (Shanahan et al., 2019). Likewise, Instagram API data evidenced only 2% of approximately three million posts disclosing mental health difficulties to post explicitly graphic images (Manikonda and De Choudhury 2017). Crucially, these images were not comprised of a short meme like caption. Furthermore, examination of the most popular (i.e., upvoted) memes related to anxiety and depression posted to the online forum Reddit failed to demonstrate any encouragement of adverse behaviour (Akram et al., 2020). Therefore, claims of excessive meme use as an indication of problematic behaviour among vulnerable individuals appear somewhat premature.

## Possible benefits of mental health memes

Members of the general population display increased positive emotional responses when observing humorous internet memes, relative to other forms of online media (Myrick et al., 2021). In the current context, a culmination of research highlights perceived benefits associated with online interaction with mental health memes (Akram et al., 2020; Drury, 2019; Gardener et al., 2021; Kariko and Anasih, 2019). Indeed, 47% of college students reported engaging with internet memes as a way of alleviating psychiatric symptoms (Kariko and Anasih, 2019). Here, contextual humour and relatability associated with dark and self-deprecating memes allowed individuals to laugh at their problems while forming a connection with others in the same situation. Indeed, previous work found poor psychological wellbeing to be related to increased sharing of mental health memes in the Spanish general population (Hernández-Cuevas and Cruz-Bermudez, 2021). The reported factors motivating such behaviour included sharing memes to alleviate tension while distracting from interpersonal struggles, and the formation of relationships with other people facing similar difficulties.

More recently, comparison of depressed individuals and controls evidenced perceptual differences in the interpretation of internet memes illustrating the experience of depression (e.g., death, suicide, isolation, hopelessness, hyper/insomnia) (Akram et al., 2020; Gardener et al., 2021). In particular, the perception of humour, relatability, shareability and mood improving potential of these memes were significantly higher among those presenting depression. Interestingly, these differences were mediated by self-defeating humour (i.e., amusing others at one’s own expense) and difficulties in using emotion regulation strategies appropriately (Akram et al., 2020; Gardener et al., 2021). These outcomes highlight potential therapeutic benefits for individuals experiencing mental health difficulties. Specifically, by facilitating a humorous take on a negative experience and situation, and the perception of peer-support through affiliation with others experiencing similar symptoms. Although memes related to depression present a dark and negative humour style, their proximal nature to those experiencing psychiatric symptoms may be considered contextually positive.

Internet memes related to non-traumatic situational work experiences are evidenced to help emergency medical service providers cope with work-related stress and burnout (Drury, 2019). Furthermore, anxious individuals observe memes related to the COVID-19 pandemic to cope with an uncertain and anxiety provoking situation (Akram et al., 2021; Myrick et al., 2021). In a sample of the US general population, Myrick and colleagues (2021) found the observation of COVID captioned memes to be associated with reduced COVID-related stress relative to those observing memes unrelated to the pandemic. Here, the authors postulate direct interaction with humorous popular media content may reduce stress and improve an individual’s emotional state. More recent evidence tentatively concludes that internet memes related to the COVID-19 pandemic may serve as a coping mechanism for individuals experiencing clinical levels of anxiety (Akram et al., 2021). In this study, the COVID-19 related memes depicted particularly negative themes relating to death and isolation. Despite this, the perception of humour, relatability, and shareability were all greater among anxious individuals relative to non-anxious controls.

The experience of psychiatric difficulties for many can be difficult to verbalise and people may feel uncomfortable disclosing the nature of their mental health difficulties, yet also feel the need to be understood and related to. Mental health memes therefore provide a wide reaching and alternate means of outlet, which allows individuals to also visualise the experience and encumbering nature of their symptoms (Akram et al., 2020). As perceived social support through online interaction appears beneficial in reducing psychiatric symptoms (LaRose et al., 2001), internet memes may act as a conduit whereby individuals form supportive socioemotional bonds with others. Together, these outcomes suggest that interacting with internet memes may help people cope with psychological distress.

## Adverse effects of mental health memes

Despite the evidenced benefits associated with mental health meme interaction, several studies highlight possible adverse consequences. First, acute exposure to mental health memes appears to increase state-like sensitivity towards anxiety, disordered eating, and suicidal ideation, but not depression (Gupta et al., 2021). However, in the absence of control memes and any group comparison based on psychiatric symptom status, these outcomes should be taken with caution. Next, employing a phenomenological analysis of memes related to suicide and death, the observation of such memes possibly obscure and trivialise the reality and inevitability of death (Manikonda and De Choudhury, 2017). However, this methodological approach may be considered as somewhat limited, relying largely upon the authors interpretation of selective data. Finally, sampling healthy adults, Akil and colleagues (2022) evidenced a reduced mood state following the observation of depressive memes when compared with neutral non-meme images. While a statistically small effect was observed (*P* = 0.045), the lack of therapeutic effect among individuals free from psychiatric difficulty remains somewhat interesting.

## Might contextual humour facilitate emotion regulation?

Humour is an essential function that facilitates the maintenance of adequate physical and psychological wellbeing (Martin, 2001). The experience of humour is frequently related to improved wellbeing, the adequate deployment emotion of regulation strategies, and the ability to cope with stressful life events (Kugler and Kuhbandner, 2015; Labott and Martin, 1987; Martin et al., 2003; Martínez-Martí and Ruch, 2014; Overholser, 1992; Ruch and Hofmann, 2017; Sliter et al., 2014).

Not all individuals observe and share humorous memes related to their specific difficulties. Indeed, individuals presenting deficits in emotion regulation are significantly more likely to interact with internet memes related to their mental health condition (Akram et al., 2020, 2021). This makes sense when considering emotion regulation difficulties predict social media use in the occurrence of a negative mood state (Drach et al., 2021). When used productively, social media can be used to upregulate positive (e.g., viewing enjoyable posts and pictures) and downregulate negative emotions (e.g., reading posts related to coping with depression).

However, mental health difficulties are related to individual differences in the strategic approach taken when using humour to facilitate cognitive reappraisal (i.e., reinterpretation of an emotion-eliciting situation to alter meaning and emotional impact) (Liu et al., 2019; Lazarus and Alfert, 1964). Typically, these differences are considered deficits in the affective (i.e., blunted humour ratings) and cognitive (i.e., impaired accuracy) aspects of humour processing (Samson and Gross 2012; Uekermann et al., 2007). However, it is possible that individuals experiencing psychological distress favour dark and negative humour due to the increased salience of their specific symptom experience (Akram et al., 2020). For example, depressed individuals prefer a comparative approach where an experienced event or situation is downplayed with another more extreme scenario (e.g., it could be worse). In contrast, non-depressed individuals prefer to focus on positive situational factors (e.g., accomplishments of the day). In this context, when used adaptively, mental health memes might diminish the meaning of certain events (i.e., perspective placement) while allowing the individual to make light of a negative experience (i.e., positive reappraisal). With that in mind, eye-tracking data indicates that the visual attention of depressed individuals were more easily captured by memes related to their symptom experience, relative to neutral control memes (Akram et al., 2021b).

Internet memes may be considered as an inside joke, frequently shared by likeminded individuals who know how to use the meme in the correct context (Zappavigna, 2012). Therefore, sharing and observing internet memes related to an individual’s particular mental health difficulty may facilitate adaptive emotion regulation strategies in those with deficits in the ability to deploy such strategies. In contrast, healthy adults free from psychiatric difficulty likely fail to understand or relate to mental health memes. In turn, this perceptual disconnect may contribute to the scepticism surrounding the use of such memes.

## Considerations moving forward

The examination of mental health memes remains at an early stage. As such, several methodological considerations should be addressed in future work. Only a handful of studies have sampled a population of individuals experiencing psychiatric symptoms in comparison to healthy controls (Akram et al., 2020, 2021; Gardener et al., 2021; Hernández-Cuevas and Cruz-Bermudez, 2021). Rather, most studies sample members of the general population and healthy adults in the absence of any experimental group comparisons. In addition, substantial variation in stimuli used limits generalisability and face validly across studies. However, the existing data should first be replicated among clinical populations, and across additional psychiatric conditions.

When extracting mental health related and neutral memes as stimuli, a systematic approach should ensure each image accurately depicts the symptom experience of the examined condition. In addition, the nature of yielded memes should remain topically and culturally relevant at the time the research is conducted. Here, the inclusion of a pilot rating study may precede the experimental protocol. Alternatively, preliminary analysis may facilitate the exclusion of unreliable or invalid stimuli. Furthermore, the inclusion of adequate neutral control memes largely remains overlooked. Rather, many studies either fail to include neutral memes or opt for generally neutral non-meme images. More crucially, much of the evidence to date appears to be based on ratings of a very small number of atypical memes. Specifically, the use of pictorial stimuli that fails to conform to the most current format of popular internet memes. Finally, additional control factors may include an individual’s frequency and exposure to mental health memes, and the severity of the content displayed in these memes.

Several studies explore the role of possible mediating factors when examining perceived benefits of mental health memes (i.e., humour style, emotion regulation: Akram et al., 2020; Gardener et al., 2021). However, additional exploration of psychosocial processes underlying these perceived benefits is required. In conjunction, comparison of individuals with and without psychiatric symptoms remains equally vital. Under these conditions, we may better understand candidate factors that may play a crucial role in addressing moderating questions such as ‘when’, ‘for whom’ and ‘under which’ conditions are mental health memes potentially beneficial for those experiencing psychiatric difficulties.

Qualitative insight from individuals with lived experience of psychiatric difficulties would gain an in-depth perspective of perceived benefits and adverse consequences associated with mental health meme interaction.

Moving beyond secondary phenomenological and cross-sectional analysis of meme ratings, a shift towards experimental techniques may yield objective insight into the role of mental health memes. To that end, eye-tracking, and brain reactivity paradigms (i.e., electroencephalography and event related potentials) approaches have preliminary assessed gaze behaviour and brain reactivity in response to the observation of internet memes related to depression.

Certainly, eye-tracking allows for visual attention to be continually recorded through the duration of stimulus presentation, expanding on snapshots provided by perceptual ratings of internet memes. As individuals experiencing psychiatric symptoms may engage with mental health memes outside of the laboratory setting, a more naturalistic approach should be taken moving forward (i.e., eye movement examination of memes embedded into webpages).

It is well established that the amygdala regulates behavioural responses to socially ambiguous signals. Across major psychiatric conditions, altered amygdala connectivity with brain regions related to emotional processing are consistently evidenced in response to disorder-congruent stimuli (Abler et al., 2007; Bermpohl et al., 2009; Huang et al., 2012; Price and Drevets 2010). In affective disorder patients, structural prefrontal cortex and functional amygdala abnormalities perpetuate connectivity to sensory cortices and regions dedicated to emotion regulation (Huang et al., 2012; Peng et al., 2019). Here, reduced prefrontal cortex volume facilitates hyperarousal of the emotion regulation system, potentiating inadequate emotional reactivity in response to aversive cognitive processes (e.g., worry and rumination) (Peng et al., 2019). Similar consequences related to the hyperarousal of the psychomotor system appear to be related to alterations in amygdala activity in patients observing disorder-congruent stimuli when compared with healthy controls (Huang et al., 2012; Kim et al., 2017; Peng et al., 2019). In the current context, it is important to determine whether the neural processing of mental health memes accentuate cortical hyperactivity in key brain regions in those experiencing psychiatric difficulties.

Internet memes related to psychiatric difficulties provide more proximal stimulus related to the symptom experience of the condition assessed, expanding beyond words, image scenes, and emotional faces. Future work should prioritise integration of experimental methods paired with perpetual ratings of well validated meme sets and psychometric evaluation of cognitive mediators.

## Conclusion

Evidence to date fails to demonstrate that mental health memes graphically visualise and promote adverse behaviour (Akram et al., 2020; Hernández-Cuevas and Cruz-Bermudez, 2021; Kariko and Anasih, 2019; Shanahan et al., 2019). Rather, social media pages dedicated to mental health memes appear to facilitate the expression of difficult emotions in a novel and creative way, providing social and emotional bonds with others, which may be perceived as socially supportive (Akram et al., 2020, 2022; Drury, 2019; Gardener et al., 2021; Kariko and Anasih, 2019). Indeed, these memes appear to be well received in those with psychiatric symptoms (albeit specific to anxiety and depression) who perceived them as humorous, relatable and a means of social connection (Akram et al., 2020, 2021; Gardener et al., 2021; Kariko and Anasih, 2019). Perhaps more surprisingly, engaging with mental health memes may present potential benefits for those experiencing psychiatric difficulty. In a social context, individuals report using memes to alleviate their symptoms and laugh at their problems while forming social connections with those in the same situation (Kariko and Anasih, 2019). Moreover, for those experiencing co-occurring difficulties in emotion regulation, mental health memes may serve to potentiate cognitive reappraisal (Akram et al., 2020).

Mental health memes are an increasingly popular and unique subculture in the context of social media with many creative individuals dedicating their time by consistently updating the online meme content to follow popular culture and trending topics. This dedication maintains the popularity of memes while accomplishing the end goal of memes: to provide humorous social commentaries, which are contextually relevant to a specific target population. Despite the prevalence of mental health memes and claims of possible negative consequences, empirical exploration in relation to psychiatric difficulty remains sparse. Nevertheless, additional research is required to substantiate previous observations and explore the potential role of mental health memes as a psychological coping mechanism.

## Data Availability

No data were generated or analysed in this study.

## Footnotes

1 While social contagion refers to the viral spread of either positive or negative attitudes, behaviours, and emotions between individuals on a large scale, this process is often negatively perceived and compared with the spread of contagious diseases
